# Contagious Comments: What Was the Online Buzz About the 2011 Quebec Measles Outbreak?

**DOI:** 10.1371/journal.pone.0064072

**Published:** 2013-05-15

**Authors:** Jennifer A. Pereira, Susan Quach, Huy Hao Dao, Jeffrey C. Kwong, Shelley L. Deeks, Natasha S. Crowcroft, Sherman D. Quan, Maryse Guay, On behalf of the Public Health Agency of Canada/Canadian Institutes of Health Research Influenza Research Network (PCIRN) Program Delivery, Evaluation Group

**Affiliations:** 1 Public Health Ontario, Toronto, Canada; 2 Département des sciences de la santé communautaire, Université de Sherbrooke, Longueuil, Canada; 3 Dalla Lana School of Public Health, University of Toronto, Toronto, Canada; 4 Institute for Clinical Evaluative Sciences, Toronto, Canada; 5 Department of Family and Community Medicine, University of Toronto, Toronto, Canada; 6 University Health Network, Toronto, Canada; 7 Laboratory Medicine and Pathobiology, University of Toronto, Toronto, Canada; 8 Institut national de santé publique du Québec, Longueuil, Canada; 9 Centre de recherche de l’Hôpital Charles LeMoyne, Longueuil, Canada; University of Ottawa, Canada

## Abstract

**Background:**

Although interruption of endemic measles was achieved in the Americas in 2002, Quebec experienced an outbreak in 2011 of 776 reported cases; 80% of these individuals had not been fully vaccinated. We analyzed readers’ online responses to Canadian news articles regarding the outbreak to better understand public perceptions of measles and vaccination.

**Methods:**

We searched Canadian online English and French news sites for articles posted between April 2011 and March 2012 containing the words “measles” and “Quebec”. We included articles that i) concerned the outbreak or related vaccination strategies; and ii) generated at least ten comments. Two English and two bilingual researchers coded the unedited comments, categorizing codes to allow themes to emerge.

**Results:**

We analyzed 448 comments from 188 individuals, in response to three French articles and six English articles; 112 individuals expressed positive perceptions of measles vaccination (2.2 comments/person), 38 were negative (4.2 comments/person), 11 had mixed feelings (1.5 comments/person), and 27 expressed no opinion (1.1 comments/person). Vaccine-supportive themes involved the success of vaccination in preventing disease spread, societal responsibility to vaccinate for herd immunity, and refutation of the autism link. Those against measles vaccination felt it was a personal rather than societal choice, and conveyed a distrust of vaccine manufacturers, believing that measles infection is not only safe but safer than vaccination. Commenters with mixed feelings expressed uncertainty of the infection’s severity, and varied in support of all vaccines based on perceived risk/benefit ratios.

**Conclusion:**

The anti-vaccine minority’s volume of comments translates to a disproportionately high representation on online boards. Public health messages should address concerns by emphasizing that immunization is always a personal choice in Canada, and that the pharmaceutical industry is strictly controlled. Illustrating the dangers of measles through personal stories, rather than scientific data only, may also serve to strengthen messaging.

## Introduction

Measles is an extremely contagious respiratory disease characterized by a fever and maculopapular rash. [1] The World Health Organization (WHO) has estimated that in 2010 there were 139,300 measles deaths globally. [1] In 2002, the Americas had successfully eliminated measles transmission by implementing a vaccination strategy that included a second dose of the vaccine to ensure high population immunity. [2] Since then, only small numbers of imported and import-related measles cases have been reported in Canada; outbreaks occurred mainly in isolated groups with limited secondary transmission in the general population. [3] However, in April 2011, the Canadian province of Quebec (population of 7.9 million in 2011) [4] experienced the largest measles outbreak in two decades, resulting in 776 reported cases, of which 80% had either not been vaccinated at all or had not received two doses of vaccine. [5] As a result, Quebec launched a school-based measles vaccination program for students and staff who were neither fully vaccinated nor naturally immune due to previous infection. However, the overall success of programs such as these is likely to be strongly influenced by public perceptions of vaccines and the diseases they protect against.

Traditionally, surveys and qualitative methods have been used to collect attitudinal information but the limitations of such approaches include their lack of timeliness, cost and limited generalizability. More recently, researchers have been utilizing data collected from social media outlets such as Facebook, Twitter and YouTube to analyze public perceptions of infectious disease outbreaks.[6]–[10] These online outlets provide a vast amount of real-time data which can be rapidly compiled and analyzed to understand current common opinions about vaccines.

Online media discussion forums are an additional source of data to gauge public perceptions of news events, as they allow readers to provide thoughts about news articles in an anonymous environment. Individuals can participate without providing personal information, which can encourage honesty without fear of the social acceptability of their comments. [11] Yet, to date, few studies have analyzed online media discussion forums to explore perceptions of vaccinations. [12].

During the measles outbreak in Quebec, several articles appeared on popular Canadian news websites to report related details, and provided readers with the opportunity to post comments in response. We evaluated these comments, with the objective of exploring public perceptions of measles and measles vaccination, during and following the outbreak.

## Methods

### Data Sources

To identify news articles regarding the Quebec measles outbreak from a variety of media sources, we searched the following national and local Canadian online English and French news sites:

English sites:

- www.cbc.ca (Canadian Broadcasting Corporation)
- www.ctvnews.ca (Canadian Television Network)
- www.globeandmail.com (Globe and Mail)
- www.nationalpost.com (National Post)

French sites:

- www.journaldemontreal.com (Journal de Montréal)
- www.lapresse.ca (Cyberpresse)
- www.ledevoir.com (Le Devoir)
- www.metro.ca (Metro)
- www.radio-canada.ca (Radio-Canada)
- tvanouvelles.ca (Téléviseurs associés)

We considered news articles posted between April 2011 and March 2012, the start date of the epidemic and the end date of the school vaccination campaign, respectively. Articles were included if they contained the words *“measles” (“rougeole”) and “Quebec” (“Québec”)*, and were reviewed to ensure that they i) predominantly concerned the outbreak, reactions or related vaccination strategies; and ii) generated at least ten readers’ comments.

### Analysis

#### Descriptive analysis

We summarized descriptive statistics for each article, including number of comments per individual. At least two researchers assessed each comment, and reached consensus on its sentiment towards measles vaccination: vaccination-supportive (“positive”), anti-vaccination (“negative”), mixed feelings (“mixed”), or a neutral stance (“neutral”). We compared comments in English and French media based on the percentage in each of these four categories.

#### Qualitative analysis

Two bilingual researchers (H.H.D and M.G) coded all of the unedited French comments independently, comparing coding lists periodically throughout the process, to ensure consistency. The French comments were translated into English, and a third researcher (J.A.P) coded the translations and conferred with H.H.D and M.G to finalize a single coding dictionary.

S.Q and J.A.P co-coded 20% of the unedited English comments independently using this dictionary, adding codes as required. After conferring and reaching consensus on the finalized coding list, they each coded half of the remaining 80% of English comments.

Following coding, the research team reviewed the results together to ensure that both clinical and methodological perspectives were brought to the analysis which was conducted in QSR NVivo® 9. Queries were run to allow data themes to emerge. Themes and subthemes were further examined by comment language, to note potential differences in topics and overall sentiment.

#### Comment approval evaluation

For news sites which allowed readers to indicate their approval or disapproval for a comment, we calculated a net approval score by subtracting the number of “dislikes”/”disapproves”/”disagrees” from the number of “likes”/”approves”/”agrees” (i.e., negative minus positive comments). We compared the popularity of the French comments vs. English comments, by ranking comments based on their net approval scores.

## Results

**Figure 1:**
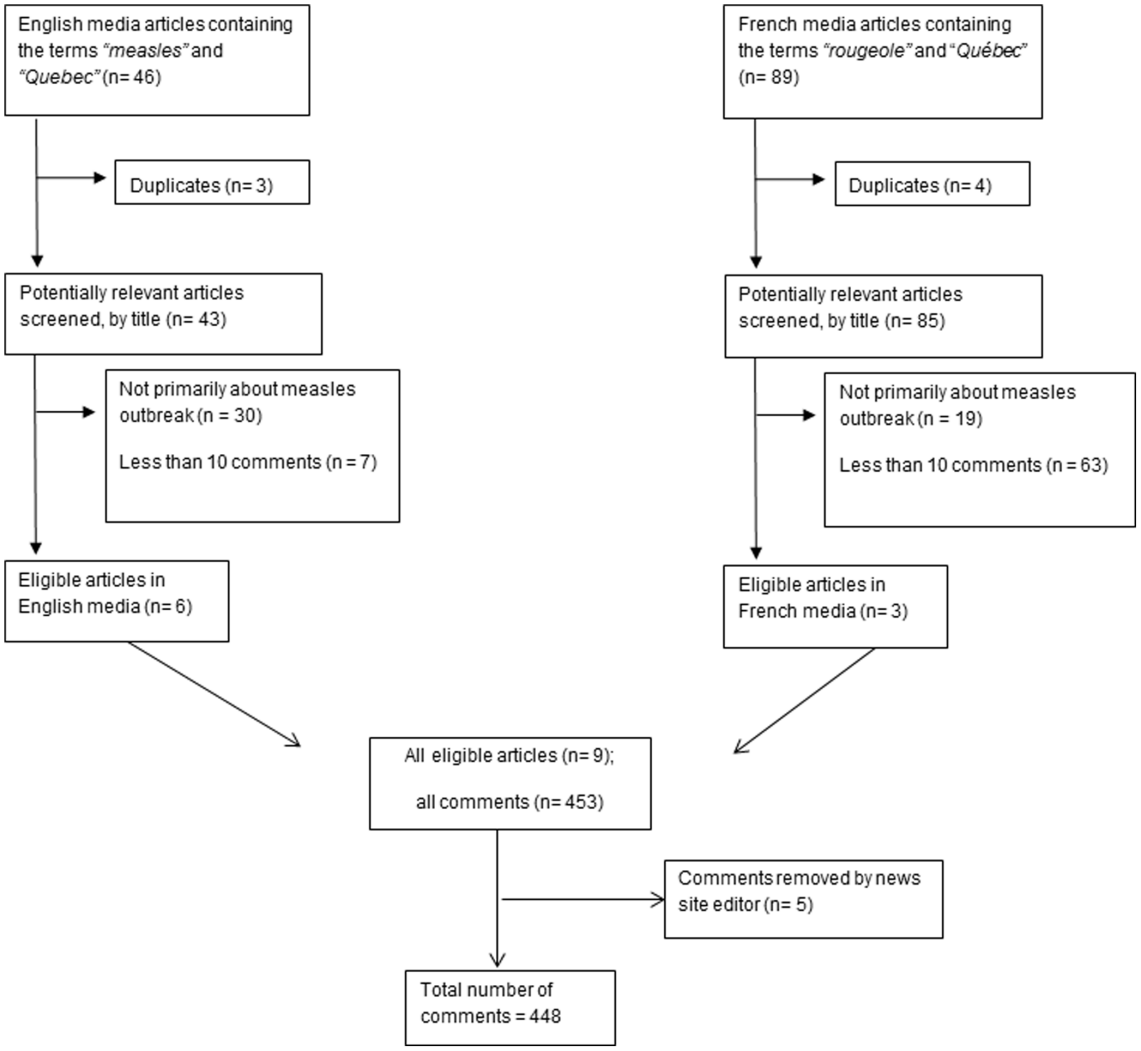
Flow diagram of inclusion and exclusion of articles. This diagram describes the search and filter process used to identify the articles that met our pre-determined inclusion criteria.

**Figure 2:**
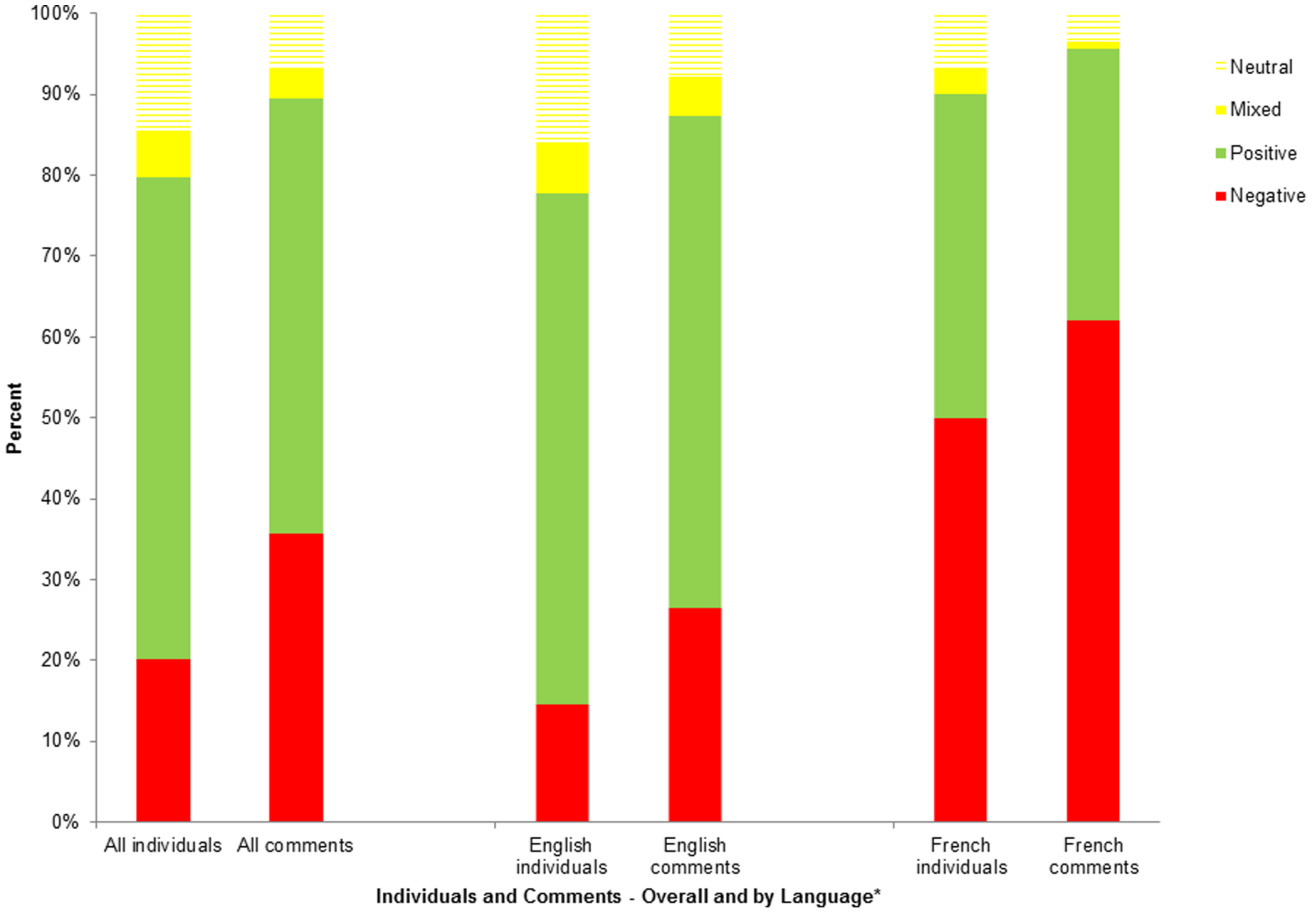
Percentage of individuals and comments, by vaccination sentiment, overall as well as per language. This describes the percentage of individuals as well as comments by vaccination sentiment (positive/negative/mixed/neutral), across all articles, and stratified by language.

Our online searches of media sites identified nine articles which met our inclusion criteria (Figure 1). From these articles, we analyzed 448 comments from 188 individuals (2.4 comments/person), in response to three French articles and six English articles; 112 individuals expressed positive perceptions of measles vaccination (2.2 comments/person), 38 were negative (4.2 comments/person), 11 had mixed feelings (1.5 comments/person), and 27 were neutral (1.1 comments/person). We further stratified by language, and found a higher proportion of negative French than English comments overall (Figure 2).

**Figure 3:**
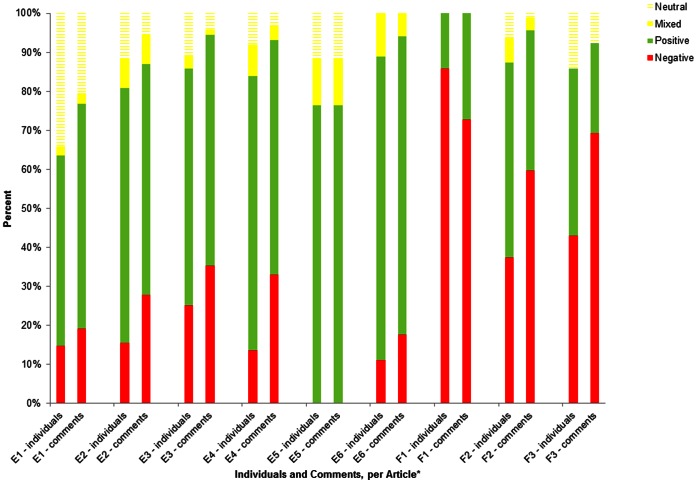
Percentage of individuals and comments, by vaccination sentiment, per article. *E1 (English article #1) = http://www.cbc.ca/news/health/story/2011/11/03/ottawa-measles-survey-parents.html. E2 (English article #2) = http://www.cbc.ca/news/canada/montreal/story/2011/10/27/mtl-measlesoutbreak.html. E3 (English article #3) = http://www.cbc.ca/news/health/story/2011/10/20/measles-quebec-vaccine-schedule.html. E4 (English article #4) = http://www.theglobeandmail.com/life/health/new-health/andre-picard/the-return-of-measles-where-did-we-go-wrong/article2052432/. E5 (English article #5) = http://www.ctv.ca/CTVNews/TopStories/20110608/measles-quebec-canada-110609/. E6 (English article #6) = http://montreal.ctv.ca/servlet/an/local/CTVNews/20110606/mtl_measels_110606?hub=MontrealHome. F1 (French article #1) = http://www.radio-canada.ca/nouvelles/sante/2011/06/07/001-rougeole-eclosion-explications.shtml. F2 (French article #2) = http://www.radio-canada.ca/nouvelles/societe/2011/10/27/001-quebec-rougeole-vaccin.shtml. F3 (French article #3) = http://www.radio-canada.ca/nouvelles/sante/2011/06/06/003-rougeole-situation-quebec.shtml.

The articles varied considerably in terms of the average number of comments generated per individual (Table 1). At the lower end, the CTV news article generated 17 comments from 17 different individuals, while conversely the October 27^th^, 2011 article on the Radio-Canada site led to an average of 5.8 comments/person. When we evaluated the comments per article, we found that the French articles had a higher anti-vaccination to pro-vaccination comment ratio than those for English articles (2.3∶1 vs. 0.35∶1, respectively; Figure 3).

**Table 1:** Eligible Canadian news articles on Measles Outbreak in Quebec – Number of Comments and Individuals.

Date	Article Title	Weblink	Number of comments	Number of comments/individual
*English articles (total number of comments = 332*)				
3-Nov-11	Quebec measles outbreak concerns Ottawa	http://www.cbc.ca/news/health/story/2011/11/03/ottawa-measles-survey-parents.html	73	1.8
27-Oct-11	Quebec battling major measles outbreak	http://www.cbc.ca/news/canada/montreal/story/2011/10/27/mtl-measlesoutbreak.html	54	2.1
20-Oct-11	Measles among vaccinated Quebeckids questioned	http://www.cbc.ca/news/health/story/2011/10/20/measles-quebec-vaccine-schedule.html	71	2.5
8-Jun-11	The return of measles: Where didwe go wrong?	http://www.theglobeandmail.com/life/health/new-health/andre-picard/the-return-of-measles-where-did-we-go-wrong/article2052432/	100	2.7
9-Jun-11	Quebec measles outbreak couldspread, warns expert	http://www.ctv.ca/CTVNews/TopStories/20110608/measles-quebec-canada-110609/	17	1
6-Jun-11	Quebec health official calls for vaccinations as 254 measles cases reported	http://montreal.ctv.ca/servlet/an/local/CTVNews/20110606/mtl_measels_110606?hub=MontrealHome	17	1.9
*French articles (total number of comments = 116)*				
28-Nov-11	Le Québec aux prises avec une épidémie de rougeole	http://www.radio-canada.ca/nouvelles/sante/2011/06/07/001-rougeole-eclosion-explications.shtml	11	1.6
27-Oct-11	Québec lance une campagne de vaccination contre la rougeole	http://www.radio-canada.ca/nouvelles/societe/2011/10/27/001-quebec-rougeole-vaccin.shtml	92	5.8
06-Jun-11	Rougeole : la Santé publiquerappelle l'importance de lavaccination	http://www.radio-canada.ca/nouvelles/sante/2011/06/06/003-rougeole-situation-quebec.shtml	13	1.9

### Comment Themes

#### Vaccine-supportive themes

Of the 112 commenters who expressed pro-vaccination beliefs in 241 posts, 24 (21.4%) included statistics or a link to a reference regarding vaccination. Of the ten individuals who posted links to sites or references to other reading, seven were referencing government or medical institution sites on vaccination. Four commenters (3.6%) either identified themselves as healthcare professionals/biologists/scientists, or related the opinions of healthcare professionals to support their arguments, while eight individuals (7.1%) shared personal experiences regarding vaccination and/or vaccine-related adverse events. Vaccine-supportive themes are explored more fully below:

Preventing disease and transmission: Commenters discussed the value of vaccination in preventing diseases which were previously a significant contributor to mortality or morbidity. They maintained that vaccination had been a key factor in improving societal health, extending lifespans, and eradicating disease. Some argued that since the majority of the population is vaccinated against measles, and only a very small number were infected during the outbreak, this actually proved the effectiveness of the vaccine, since a far smaller proportion were not vaccinated, and many of these individuals became infected with the virus.

Severity of diseases: Several individuals commented on the severity of measles, acknowledging that the majority of cases result in full recovery but that there are a small percentage of infections which cause major sequelae and even death. Five individuals had personal experience with measles, either having had it earlier in life themselves, or knowing someone who was impacted by the disease. These commenters expressed disbelief and frustration that anyone would refuse to be vaccinated, given the potential for serious health consequences.

Societal responsibility: Many commenters expressed the importance of getting vaccinated to protect those with weakened immune systems, as well as those for whom vaccination was not effective. These individuals felt that vaccination was a societal responsibility that trumped one’s right to choose. Many discussed the importance of herd immunity to protect others and to improve public health overall.

Vaccine benefits outweigh risks/refutation of link to disease: Several vaccine-supportive individuals stated that while the benefits of vaccination were evident and well-proven, in the form of decreased infections and fewer sequelae, no link between vaccine and disease had ever been founded. Others specifically named the Andrew Wakefield scandal, commenting that the link between autism and the measles-mumps-rubella vaccine had been discredited. A small number of individuals did believe that vaccinations may lead to diseases such as attention deficit hyperactivity disorder (ADHD) or autism, but also felt that these cases were few and far between, and that given the success of the vaccine and the severity of measles, such risks were warranted. Those who discussed toxins either denied that vaccines contain thimerosal or other “poisons”, or indicated that the amount of such a substance in a vaccine is lower than is present in common foods and household goods.

Anti-vaccination movement: A number of individuals described the anti-vaccination movement as preying on confused, frightened and well-intentioned parents, and supported by those with monetary motives. There were many comments regarding information source, and specifically the credibility of medical institutions, physicians, and public health officials versus celebrities and others without a strong scientific background. Those supporting vaccinations often reinforced their statements with statistics from public health websites, journal articles, or newspaper quotes, and felt that the spokespeople for the anti-vaccination movement were celebrities and others who should not be viewed as appropriate sources. They also blamed parents and caregivers for the outbreak, describing it as an easily preventable drain on the healthcare system.

*“Hope all the “know-it-all” parents who decided to have their children forgo their vaccinations are happy. This is what happens when uninformed people think they can make an informed decision. As they say” a little information is more dangerous than none at all”.”*

*-Marge, in response to* http://www.ctv.ca/CTVNews/TopStories/20110608/measles-quebec-canada-110609/

#### Anti-vaccination themes

Thirty-eight individuals posted 160 comments indicating that they were against measles vaccination. Ten (26.3%) referenced statistics or a website to support their argument, two citing government or medical websites, three citing anti-vaccination websites, and two referencing other articles in the press. Six (15.8%) indicated that they were healthcare professionals/biologists/scientists, or relayed the opinion of someone who was, while five individuals (13.2%) shared personal stories regarding measles, vaccination and/or vaccine-related adverse events. Common themes are described below.

Conspiracy: Individuals described a mistrust of pharmaceutical companies, stating that they were either i) intentionally providing faulty vaccines to worsen the public’s health and create a dependence on medications; or ii) manufacturing vaccines which were ineffective or unsafe to make money. Two commenters wrote that they knew people who worked at these companies, who did not allow their own families to get vaccinated. Others wrote about conspiracies with physicians and public health, stating that they were linked to pharmaceutical companies and therefore pushing the manufacturers’ agendas, or working with government to spark fear in the public.

Safety: Safety of vaccines was discussed, for measles as well as other infectious diseases, particularly human papillomavirus (HPV). Several commenters described vaccines as full of thimerosal, “toxins”, “poisons”, and mystery ingredients that have not been disclosed to the public, and which they felt were not worth injecting into their bodies to avoid an infection that they assumed to be relatively harmless. Several discussed mycosis, fatigue, autism, ADHD, liver issues, and other health issues they felt were caused by the vaccine. Individuals expressed the belief that even if the vaccine was effective, it was preventing a very rare disease, at the risk of exposing society to new health problems.

Efficacy: The measles outbreak in Quebec sparked much debate about the vaccine’s efficacy. Many commenters provided related statistics, including the number who had been vaccinated and the number who had not. Because several of those who were infected were individuals who had been vaccinated against measles, some believed this indicated a less-than-effective vaccine. These individuals felt that if the vaccine truly worked, none of those infected during the outbreak would have been previously vaccinated.

H1N1: The 2009 pandemic coloured many commenters’ opinion of vaccination in an unfavorable way. There was sentiment that the H1N1 vaccination campaign had been a scam to frighten the public and boost vaccine sales for drug manufacturers, eroding trust in all public health efforts for some commenters. There was also discussion about conspiracies between government and vaccine industries to spread fear about the pandemic, and increase vaccine sales. Additionally, several commenters wrote about having acquired H1N1 or knowing someone who had, and finding it to be a very mild version of influenza, rather than life-threatening as reported; this has also affected their impressions of the severity of other infections.

*“During the widespread paranoia over H1N1, my family got this infamous flu, which was being described as apocalyptic[…]. Well, none of us died – we aren’t crazy, we took the necessary precautions to not contaminate anyone else, and we are all very alive today. I have the feeling that this new vaccination campaign is part of the ‘trend’ of making people who prefer not to be vaccinated, for all sorts of good reasons, feel guilty, including the despicable contents of the vaccination itself, as well as the campaign of fear that began with H1N1. I won’t give in to either.”(translated from French)*

*-Marianne Longland Marianne, in response to*

http://www.radio-canada.ca/nouvelles/societe/2011/10/27/001-quebec-rougeole-vaccin.shtml

Freedom of choice: Anti-vaccination commenters felt that the choice to get vaccinated should be up to the individual or their caregiver, and that vaccination was not an issue of social responsibility. Since this opinion was aligned with that of vaccines being unsafe, individuals felt that they should not be risking harm to themselves for the good of the public. They also felt that the vaccination campaign’s goal was to frighten or guilt the public into getting vaccinated, and that no one should be concerned as to what others did, because those who were vaccinated would be protected if the vaccine truly worked. When faced with the argument that vaccination was important to protect those who could not be vaccinated due to contraindications or a weakened immune system, or those for whom the vaccine proved ineffective, they argued that those individuals should not rely on others to get vaccinated, but should instead take all necessary precautions such as wearing facemasks, avoiding areas where infections are more rampant, and hygienic measures.

Alternatives to vaccination: Commenters discussed vaccination as an immune system-suppressing act that should be replaced with other health prevention measures such as hand-washing, proper hygiene, vitamin intake, etc. The sentiment was the vaccination was harmful to the body and that if someone was healthy, their immune system would be capable of fighting off an infection using less invasive measures. Additionally, even if the person got the infection, it would be benign and would prevent future recurrences by giving that individual lifelong immunity.

#### Mixed feelings regarding measles vaccination

Eleven individuals posted 17 comments describing mixed feelings regarding measles vaccination, viewing the issue as less than straightforward. Of these individuals, one referred to statistics to strengthen their point, one identified themself as a healthcare professional, and three described personal experiences with vaccination or related adverse events.

Those with mixed feelings towards measles vaccines expressed uncertainty about the infection’s severity, and varied in support of all vaccines based on perceived risk/benefit ratios. For instance, they were unclear as to whether the severity of the measles infection warranted vaccination, but felt that there might be other diseases serious enough to vaccinate against. These commenters spoke of over-vaccination, or the notion that while they did see the importance of vaccines in preventing disease and saving lives, society was now all too eager to vaccinate for every possible disease or infection, and that they only believed in vaccinating for certain diseases which they deemed serious enough.

#### Neutral opinion of measles vaccination

Twenty-seven individuals expressed a neutral stance towards measles vaccination in 30 separate comments. This group did not indicate any specific views on the outbreak, the severity of measles or vaccination campaigns, but instead used the boards to ask questions such as what is measles, how does one know if they are immune, and the merits of passive versus active immunity.

### Comment Approval Rating

Of the nine articles for which readers’ comments were reviewed, seven were from sites that allowed readers to indicate their support or lack thereof with a comment’s sentiment, by clicking “like”/”approve”/”agree” or “dislike”/”disapprove”/”disagree”. The three comments with the highest net approval scores were each vaccine-supportive in sentiment. The comments collectively defend the importance of the measles vaccine and blame the outbreak on the anti-vaccination movement for providing the public with false information regarding the vaccine’s safety:

“*The MMR vaccination is very important. You don’t vaccinate to protect yourself or your children, you vaccinate to protect the population. New parents out there, please trust your doctors and scientists and not fringe celebrities and online sites with no credibility!!” (+96)*

*-the pags, in response to* http://www.cbc.ca/news/health/story/2011/11/03/ottawa-measles-survey-parents.html

*“Way to go, anivaxxers [sic]. You’re succeeding in convincing the gullible that vaccination is bad, despite volumes of carefully controlled clinical trials that prove the opposite. And now a large group of children are getting needlessly sick because of you. Shame on you.” (+113)*

*-ScienceBoy, in response to*

http://www.cbc.ca/news/canada/montreal/story/2011/10/27/mtl-measlesoutbreak.html

*“Wow, so much damage from one (now non-MD) who tried to scare people into believing vaccines are dangerous. All so he could make money. Of course the medical journal that published the BS is also culpable.” (+143)*

*-Robertwager, in response to*

http://www.cbc.ca/news/canada/montreal/story/2011/10/27/mtl-measlesoutbreak.html

The three comments with the lowest net approval score each expressed anti-vaccination sentiments. The commenters described a distrust of vaccinations, concerns regarding their efficacy and safety, as well as notions of pharmaceutical company conspiracies to provide vaccines at the risk of public harm in order to increase profits:

*“Yet another excuse to vaccinate anything with a pulse…not letting my kid be their guinea pig.” (-163)*

*-Paco514, in response to* http://www.cbc.ca/news/canada/montreal/story/2011/10/27/mtl-measlesoutbreak.html

*“Many children who catch measles have already been vaccinated* http://www.naturalnews.com/033399_vaccines_measles.html. *Many children who receive vaccines go into shock as well. Big PHARMA wants you vaccinated more than anyone else. I don’t listen to quacks like the rest of the sheeple.” (-162)*

*-Paco514, in response to* http://www.cbc.ca/news/canada/montreal/story/2011/10/27/mtl-measlesoutbreak.html

*“Vaccines… LOL Vaccines #1 job is to Destroy the immune system, so you have a long suffering life, relying on Big Pharma for relief…….sorta. Haven’t you people realized yet, that Big Pharma makes More money, when More people are sick?…there is not Compassion in Big Pharma….only greed.” (-99)*

-*Jgoyum, in response to* http://www.cbc.ca/news/health/story/2011/10/20/measles-quebec-vaccine-schedule.html#socialcomments

The comments with the highest and lowest net approval scores were all from English media sources. When we examined the three French comments with the highest net approval scores, we found that the top two were supportive of vaccinations.

*“I won’t go back over the whole debate on the dangers of vaccination. The risks are known and they are well-controlled. We shouldn’t question everything because of the doubts harboured by the anti-vaccination conspiracy theorists.*

*1) There is more mercury in a can of tuna than in a dose of vaccine…*

*2) Not only have the alleged neurological consequences of vaccination, such as autism and hyperactivity, never been proven by serious scientific studies, they have been refuted repeatedly. The medical journal that published the only article claiming to prove this causality has published a retraction.*

*3) We may criticize vaccination campaigns but we can’t lump them all together: the HPV, H1N1, and MMR vaccines do not all have the same level of safety or efficacy nor do they have the same history or the same advocates.*

*4) No, vaccination is not a panacea and yes, it has sometimes caused problems when it has not been properly administered, but these disadvantages are extremely minimal compared to the benefits for humanity.*

*5) Vaccination is a matter of public health. We all share responsibility for the health of others through our personal choices. We need to keep this in mind, too.” (+53) (translated from French)*

*-maredtoundra, in response to* http://www.radio-canada.ca/nouvelles/sante/2011/06/07/001-rougeole-eclosion-explications.shtml

*“[…] Quite the contrary, many believe that it is still possible to eradicate the disease, as was done with smallpox in 1978. I agree that the disease is, more often than not, benign, but wouldn’t it be great to eradicate it altogether?!” (+22) (translated from French)*

*-maredtoundra, in response to* http://www.radio-canada.ca/nouvelles/sante/2011/06/07/001-rougeole-eclosion-explications.shtml

However, the French comment with the third highest net approval score was from an individual who had expressed anti-vaccination views in previous comments, and who now questioned the education of those who supported vaccine use:

*“And to think that our institutions don’t teach anything…no comment!!!” (+10) (translated from French)*

*-des questions, in response to* http://www.radio-canada.ca/nouvelles/sante/2011/06/07/001-rougeole-eclosion-explications.shtml

The three French comments with the lowest net approval scores were all anti-vaccination in sentiment, describing measles as a benign infection and vaccines as unsafe, ineffective, and not needed, with the only possible exceptions being by individuals with weak immune systems.

“*The vaccine should only be administered in developing countries (where children are often very weak) or only in the case of very young children who are sick. As far as I am concerned, for a child who is healthy, it’s perfectly normal to catch measles and to get over it (cases of complications happen often with children who were already weak before they got measles). After that, you are immune for life. In our society, we often have a tendency to take the easy way out. In this case, getting vaccinated to avoid disease. We don’t trust our bodies anymore; they are designed to defend themselves. If you are healthy to start with, there really isn’t any risk. We are “programmed” to resist [disease]…” (-35) (translated from French)*

- *Marie-Joëlle Courtemanche, in response to* http://www.radio-canada.ca/nouvelles/sante/2011/06/07/001-rougeole-eclosion-explications.shtml

“…*yes, there is less mercury in a vaccine than in a can of tuna but in a vaccine, it’s in the form of thimerosal which is also 1,000 times more toxic than mercury and when you ingest mercury orally, you excrete a lot of it when you eliminate, which doesn’t happen when you are vaccinated. H1N1 vaccine, they made a “non-adjuvanted” vaccine for pregnant women that contains 100 times more thimerosal. This one fact proves beyond a shadow of a doubt that the companies, it doesn’t matter which one (they are [sic] have the same shareholders anyways) are acting in bad faith and trying to weaken the population. Personally, I felt a lot of fatigue and had difficulty concentrating after being vaccinated against meningitis and against hepatitis A & B. Two weeks later, I had generalized mycosis. If that’s the choice, it’s better to skip the vaccine and deal with the disease as a healthy person! (-27) (translated from French)*

*-283506*, *in response to* http://www.radio-canada.ca/nouvelles/sante/2011/06/07/001-rougeole-eclosion-explications.shtml

*“If you really want to understand vaccinations and their impact, read the literature: the medical mafia. They show us what they want us to see. Anyone who speaks out or who dares to say out loud what others are thinking gets lynched. Don’t forget that today’s crazy people are tomorrow’s geniuses.”(-24) (translated from French)*

*-France Paradis, in response to* http://www.radio-canada.ca/nouvelles/sante/2011/06/07/001-rougeole-eclosion-explications.shtml

## Discussion

Quebec’s 2011 measles outbreak prompted much Canadian media attention regarding those infected, as well as the public health reaction. We evaluated readers’ comments in response to French and English online news articles, to better understand public perception of the outbreak, and to assess beliefs regarding the severity of measles, and the value of vaccination against this infectious disease. Our results indicate that the majority of the online readership that responds to news articles is supportive of vaccination. However, the anti-vaccine minority is a vocal presence on online news forums, particularly in French media; the volume of comments by this small but opinionated opposition translates to a disproportionately high representation on these forums. There were several arguments presented both in support of and against vaccination, with common themes arising across English and French comments.

Commenters frequently supported their views with the use of statistics, as well as quotes from and references to books, online sites, and journal articles, indicating that these individuals’ beliefs may be beyond surface reactions, and that they have taken steps to learn more about the subject. However, the misinformation which proliferated in the comments indicates a strong need for the health community to ensure that accurate vaccination information is available to the public, through various engagement activities, including but not limited to monitoring of and participation in web forums to counter anti-vaccine arguments. The perpetuation of false information was frequent with respect to statistics; those who upheld anti-vaccination views felt that unless the vaccine was 100% effective, it was not worth taking. Many believed that since some individuals affected by the outbreak had been previously vaccinated, the vaccine was ineffective. Despite other readers rationalizing the measles incidence rate in vaccinated versus unvaccinated populations, many of those with anti-vaccination beliefs indicated that they felt the risks associated with vaccines were dire and/or frequent, and not worth accepting if there was any chance that the vaccination could be ineffective. A small number of anti-vaccination commenters expressed a familiarity with the concept of herd immunity but also indicated skepticism that it actually existed. Several comments conveyed inaccuracies regarding this concept of group protection, believing that whether they chose to get vaccinated or not had no bearing on others, and that it was a completely personal decision without any type of societal ramification. The severity of measles was also questioned. Many comments described measles as a benign infection; one confused it with chickenpox, and others described it as a common childhood affliction that previous generations had survived, that did not warrant vaccination.

Misinformation was also evident with respect to the Andrew Wakefield article in the Lancet journal which was hotly debated throughout the comments. Many commenters referred to the original article, its official withdrawal by Lancet editors, as well as the investigation by reporter Brian Deer. Those supportive of measles vaccination were often well-versed with the details of the study as well as its recant, describing it as a conspiracy against public health, and lacking any evidence of a true association between the vaccine and autism. However, those against the vaccine did not acknowledge the official withdrawal of the study, believing that such a connection existed not only with autism, but also with other conditions including attention-deficit disorders, asthma, cancer, and mycosis. Given their belief in this alleged link, they did not feel that anyone should have the right to tell them that they must be vaccinated, as the social responsibility no longer applied when one’s own life was put at risk. Anti-vaccination themes appear to comprise both inaccuracies as well as alternate perspectives regarding conventional medicine, healthcare authorities, and risk/benefit analysis; such themes have also arisen in previous studies examining the content of anti-vaccination websites. [13] It is important for public health to engage in dialogue, both online and elsewhere, to further understand these perspectives, and address the concerns from which they may be based.

Many individuals had strongly negative or positive viewpoints, engaging in discussions on the boards which frequently turned into “back and forths” between a small number of individuals. Prior research has dictated that it is difficult to change the mind of someone who has declared themselves to be against vaccination. [14] However, there is a sizable proportion of commenters who appear conflicted about their vaccination stance, and are undecided about their opinion. Of the 188 individuals who commented, 27 expressed no opinion (14.4%) while 11 had mixed feelings (5.9%). Their contributions to the online boards included questions about the vaccine, inquiries about the vaccination schedule, the severity of measles, and how to determine whether one has immunity. Those with mixed feelings often expressed a type of severity hierarchy with respect to vaccinations, justifying vaccinations against infections they judged to be life-threatening or associated with severe sequelae, but reluctant to immunize for other “minor infections”. This is a group that appears receptive to more knowledge on this topic, and in order to maximize the success of future vaccination campaigns, it is important to target this population, and make sure they are properly educated on the value of vaccination.

While there were no themes that were specific to one language only, we observed that anti-vaccination sentiment was expressed more commonly in French comments. The French commenters posted with greater frequency on average than English commenters (3.8 vs. 2.1 comments/person, respectively). The range of anti-vaccination commenters across the three French articles was 37.5–87.5% while for English comments it was 0–25%. These are based on small samples, with two of the three French articles generating less than 13 comments, while the third French article had the highest average number of comments per individual. It is possible that the measles outbreak in Quebec is reflective of this increased vaccine-negative opinion, but it is difficult to determine from this small study, and further research on public perceptions of vaccination in Quebec would be valuable to explore this potential trend. A 2011 survey of 1,745 Canadian parents on immunization issues found several key differences between Quebeckers and individuals from other provinces with respect to vaccination-related behavior. [15] Quebeckers were more likely than parents from other Canadian jurisdictions to have reported barriers that have prevented them from accessing a healthcare worker to get an immunization, and also more likely to agree that children receive too many vaccines these days. They were less likely to believe that alternative methods may eliminate the need for so many vaccines. Compared to residents of other provinces, Quebec parents are more likely to have had a discussion with a healthcare professional regarding immunizing their child, and popular resources for obtaining immunization information include the Health Canada website, their *centre local de services communautaires (community health centre)*, or their family physicians. Therefore it is particularly important that accurate and consistent healthcare information is available to parents, both online, and at public health clinics and physician offices.

This study is not without limitations. Although we attempted to be comprehensive in our search, it is possible that we may have neglected to include all media sources, particularly smaller, local news outlets, and may have missed relevant articles. Additionally, most small media sources do not allow readers to comment on their articles, and therefore it is not possible to gauge the perceptions of their readership with respect to vaccination. We do not know the demographics of those who contributed to the forums and therefore cannot whether they are truly representative of the source’s overall readership, or the Canadian population, particularly given that those who comment on online forums are typically those who have very strong views on the subject. We assumed that every unique commenter name represented a single individual but it is conceivable that individuals may have assumed different names when commenting on different articles, or perhaps even the same article. Therefore, the total number of commenters we have reported may be an overestimation. However, the anonymity was also a strength, as it likely led to more honest comments overall.

Our study has identified that readers have a range of responses with respect to the 2011/12 measles outbreak in Quebec. The majority of the comments were pro-vaccination, recognizing the potential consequences of acquiring the measles infection, and the benefits of the vaccine. However, the comments indicate that there appears to be a small but vocal opposition to vaccines, as well as a subpopulation that is vaccine-hesitant and may be open to adopting either pro- or anti-vaccine stances, given further information. To maximize the success of vaccination campaigns in preventing future measles outbreaks, it is important to limit the potential influence of anti-vaccination commenters on those still uncertain about measles vaccination by addressing their key concerns. Public health messages should be designed accordingly, emphasizing that vaccination is always a personal choice in Canada, and that the pharmaceutical industry is strictly controlled and excluded from public health decision-making, and illustrating the severity of measles through personal stories rather than scientific data only.
